# Frequency-specific photobiomodulation at theta and gamma enhances cognitive networks and mitigates age-related decline

**DOI:** 10.1007/s11357-026-02278-9

**Published:** 2026-05-04

**Authors:** Natalia Arias, Lucía Rodríguez-Fernández, Candela Zorzo, Verónica Peña León, Laura Mañas Cordero, Alba Gutiérrez-Menéndez, Juan A. Martínez, Jorge L. Arias

**Affiliations:** 1 Department of Psychology, Faculty of Life and Natural Sciences, Brain and Behavior Group, Nebrija University, Madrid, Spain; 2 Health Research Institute of the Principality of Asturias, Oviedo, Spain; 3 Neuroscience Laboratory, Department of Psychology, University of Oviedo, Oviedo, Spain; 4 INEUROPA, Institute of Neurosciences of the Principality of Asturias, Plaza Feijoo S/N, Oviedo, Spain; 5 Electronic Technology Area, University of Oviedo, Gijón, Spain

**Keywords:** Gliogenesis, Synaptogenesis, Inflammation, Apoptosis, Cognition, Ageing

## Abstract

Photobiomodulation (PBM) is a non-invasive strategy to enhance cognitive function, yet the effects of stimulation frequency remain unclear. We applied pulsed 810 nm PBM at 5 Hz or 40 Hz to the frontal cortex of adult rats, and 40 Hz PBM to aged rats. In young adults, both stimulation frequencies enhanced cognitive flexibility, and in aged rats 40 Hz PBM improved learning speed. In terms of brain changes, we studied cytochrome c oxidase (CCO) activity, c-Fos expression and protein levels. We observed a decrease in the prefrontal CCO activity in adults, and an increase in prefrontal c-Fos expression, both with 40 Hz. Regarding protein levels in young rats, 5 Hz PBM reduced pERK expression in the hippocampus and p38 in the prefrontal cortex, while regulating interleukins and cytokines in these regions. Additionally, 5 Hz upregulated Synapsin-I expression in both the prefrontal cortex and hippocampus, and increased PSD-95 levels selectively in the hippocampus, highlighting its role in synaptic plasticity and memory consolidation. Notably, 5 Hz also increased GFAP expression in both regions, and selectively upregulated NF-κB expression. 40 Hz PBM reduced pERK expression in the hippocampus and p38 in the prefrontal cortex, also modulating interleukins and cytokines in these areas. Additionally, 40 Hz increased p38 expression in the hippocampus and reduced p53 and BAX levels in the prefrontal cortex. Also, 40 Hz upregulated Synapsin-I in both regions and increased PSD-95 expression in both the prefrontal cortex and hippocampus. As observed with 5 Hz, 40 Hz also elevated GFAP expression in both regions. In aged rats, PBM reduced Iba-1 in prefrontal cortex and hippocampus, enhanced NeuN in prefrontal cortex, and decreased p38 and BCL-2 in the hippocampus. Collectively, these results demonstrate frequency-dependent modulation of neuroinflammation, synaptic plasticity, and apoptosis, while highlighting the beneficial effects of 40 Hz PBM in aged subjects, supporting PBM as a targeted approach to improve cognitive function across the lifespan.

**Graphical Abstract:**

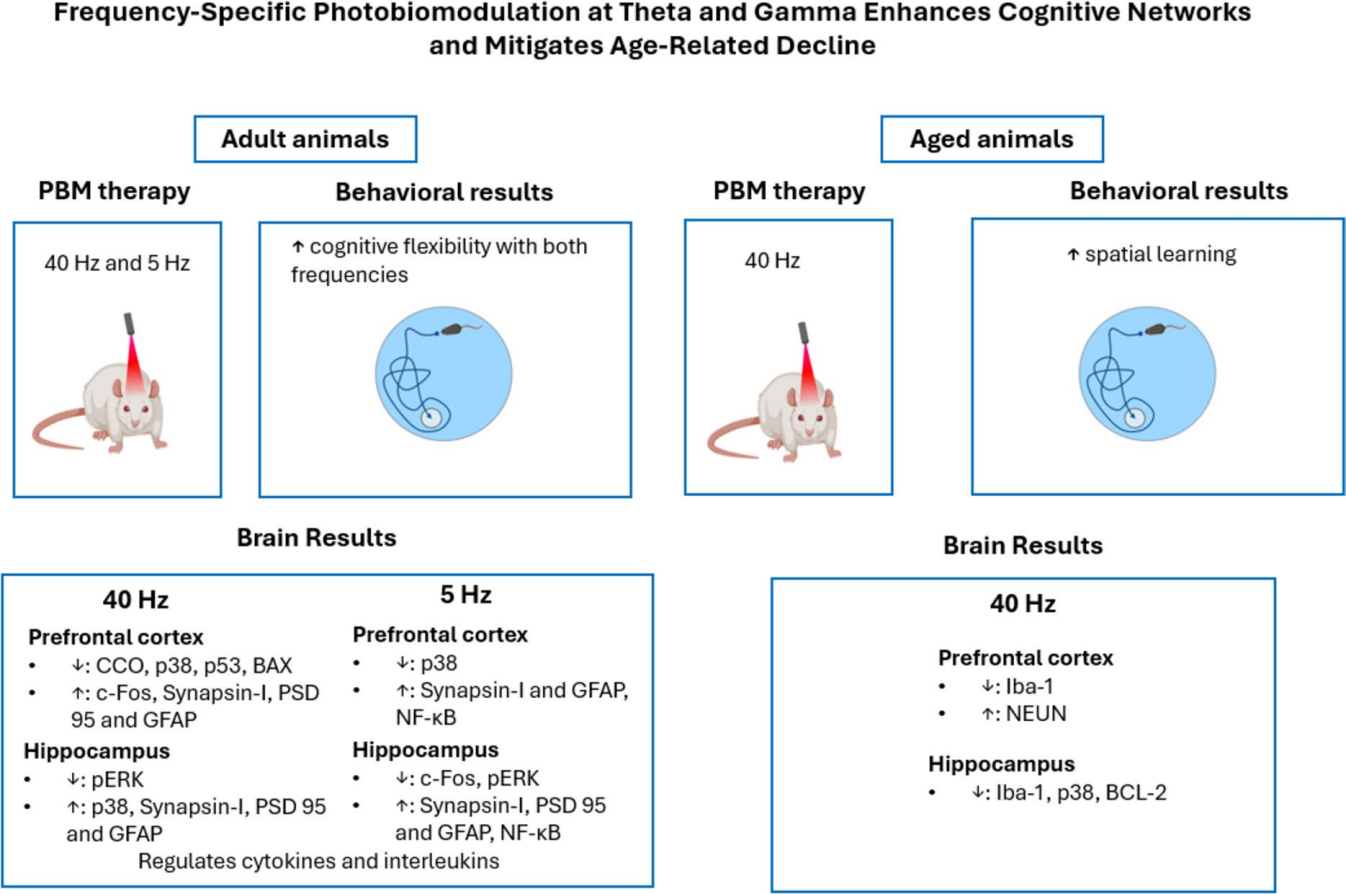

## Introduction

Photobiomodulation (PBM) applies non-thermal, non-ionizing electromagnetic radiation to stimulate biological processes [1]. It commonly uses visible or near-infrared (NIR) light (400–1100 nm) delivered through lasers, LEDs, or broadband sources [2]. Although the underlying mechanisms are not fully understood, mitochondria are recognized as key photoacceptors [3, 4]. Light absorption increases adenosine triphosphate (ATP) production, initiating secondary signaling cascades that alter redox homeostasis and regulate proliferation, differentiation, and survival [5]. Additional photoacceptors have also been proposed, broadening the range of PBM-mediated effects [6].

The mode and frequency of stimulation strongly influence PBM outcomes. Pulsed wave (PW) light is often more effective than continuous wave (CW), with deeper tissue penetration and reduced thermal load [7], making it particularly suitable for in vivo applications. Frequency-specific effects have been described at 10, 40, and 100 Hz. For example, 10 Hz stimulation reduces nitric oxide and cortisol in rodents with anxiety- and depression-like behaviors [8–10], protects against cognitive impairment after stress or sleep deprivation [11, 12], and enhances mitochondrial function. Higher frequencies, such as 40 Hz and 100 Hz, improve cognition in humans, with 40 Hz showing slightly greater benefits through modulation of neural oscillations [13–15]. Evidence of PBM-induced synaptogenesis in hippocampal and motor circuits, together with frequency-dependent modulation of neural activity, suggests that targeted PBM may help counteract mitochondrial dysfunction.

Aging is a critical context for such interventions. It is accompanied by reduced neurogenesis, impaired plasticity, demyelination, hypoperfusion, and blood–brain barrier disruption [16, 17]. Mitochondrial dysfunction, driven by impaired mitophagy and declining respiratory capacity, further compromises neuronal survival [18–21], contributing to neuroinflammation and cognitive decline. These changes highlight the need for safe, non-invasive strategies to preserve brain function across the lifespan.

Here, we examined PBM at 5 Hz and 40 Hz applied transcranially to the prefrontal cortex (PFC) of adult rats and PBM at 40 Hz applied transcranially to the PFC of aged rats. Both 5 Hz and 40 Hz stimulation improved cognition, with 40 Hz producing more immediate effects. These behavioral outcomes were accompanied by increased synaptogenesis and gliogenesis, as well as frequency-dependent modulation of neuroinflammatory responses.

Based on these findings in adult animals, 40 Hz PBM was subsequently applied to aged rats, further improved cognition and reducing age-related neuronal loss. However, as only one stimulation frequency was tested in aged animals, these results support the efficacy of 40 Hz PBM in aging rather than direct frequency-specific comparisons. Together, our findings identify frequency-specific PBM as a promising non-invasive approach to counteract cognitive decline, with evidence for frequency-dependent effects in adults and beneficial effects of 40 Hz stimulation in aged subjects.

## Methods

### Animals

For the adult study, a total of 21 male 4-month-old Wistar rats (mean weight 428.7 g at the end of the experiment) were used, and for the aging study, 14 male 18-month-old Wistar rats (mean weight 568.5 g at the end of the experiment), all obtained from the vivarium of the University of Oviedo. Rats were housed under standard laboratory conditions (20–22 ºC, 65–70% relative humidity) with a 12 h light/12 h dark cycle (lights on 08:00–20:00). Food and water were provided ad libitum, and experimental sessions were conducted during the light phase between 09:00 and 13:00.

All animal procedures were performed in accordance with Directive 2010/63/EU of the Council of the European Communities and Royal Decree No. 53/2013 of the Ministry of the Presidency on the protection of animals used for scientific purposes. The Ethics Committee of the Principality of Asturias approved the study.

Adult animals were randomly assigned to three groups: control (No PBM, n = 7), PBM at 40 Hz (n = 7), and PBM at 5 Hz (n = 7). Aged animals were randomly assigned to two groups: control (No PBM, n = 7) and PBM at 40 Hz (n = 7).

### Photobiomodulation therapy

#### Apparatus

PBM was administered using an 810 nm laser with an output power of 40 mW. The laser emitted square pulses at either 40 Hz or 5 Hz with a 50% duty cycle, depending on the experimental condition. PBM was delivered in 36 cycles, each consisting of 40 s of laser activation; due to the 50% duty cycle, the effective light exposure per cycle was 20 s, resulting in a total cumulative exposure of 720 s. The total light dose was 33 J/cm^2^. The laser beam had a spot size of 0.0495 cm^2^ and was positioned on the midline of the dorsal surface of the shaved head, in the region between the eyes and ears. Approximately 0.8% of the applied power reaches the brain tissue. This value was previously determined in rat skulls using a PM 160 optical power meter (ThorLabs, United States). Skull was placed on a bench designed for this purpose wherein at the top was the radiation laser and at the bottom, below the skull, the optical power meter.

#### Application

All animals were first handled to facilitate habituation to the researcher and the experimental procedure. The fur between the eyes was shaved to optimize light penetration over the frontal cortex. During the habituation session, the device was placed over the frontal cortex with the laser switched off, and animals were gently immobilized on a soft surface for three cycles of 4 min each (12 min total per animal).

For the treatment phase, the procedure mirrored the habituation duration. In the adult study, PBM was applied at 5 Hz or 40 Hz for five consecutive days, whereas in the aged study, PBM was applied at 40 Hz for eleven consecutive days. The laser device was turned on only for the PBM groups, while control animals underwent the same handling and positioning without device activation. PBM protocols were tailored to the age group, precluding direct quantitative comparisons across ages. Analyses were restricted to within-cohort comparisons to control for age-related differences in baseline cognition and neuroplasticity.

### Spatial learning and reversal learning

#### Apparatus

Hippocampal-dependent spatial reference memory was assessed using the Morris Water Maze (MWM). The maze consisted of a circular pool (150 cm diameter) placed in a 16 m^2^ room dimly lit with two 4000-lx lamps. Two black panels with distal spatial cues (a green hexagon on one panel; a blue triangle and an orange rectangle on the other) were positioned around the pool. The escape platform was a black cylinder (10 cm diameter), and water temperature was maintained at 22 ± 2 ºC throughout the procedure. The pool was conceptually divided into four quadrants (A–D), and animal performance was recorded with a Sony V88E video camera and analyzed using EthoVision XT 14.0 (Noldus Information Technologies, Wageningen, Netherlands).

#### Procedure

The learning protocol lasted five days. Before starting the learning protocol, animals were habituated to the task through four trials with a visible platform (2 cm above the water surface) placed at the pool center. The subsequent four days comprised the acquisition phase, with the platform hidden 2 cm below the water surface in quadrant D, and four trials per animal each day. Animals were placed in a pseudo-randomized start quadrant and given 60 s to locate the platform; if unsuccessful, they were guided to the platform and allowed to remain for 15 s. Inter-trial intervals were 30 s, during which animals were placed in a black bucket. Immediately after daily acquisition, a 30 s probe trial was performed with the platform removed to measure time spent in each quadrant. To prevent potential extinction, an additional trial with the hidden platform in quadrant D was conducted on all days except the last.

A reversal learning (Reversal, R) session was conducted on the following day, with the platform relocated to quadrant C. Four trials were conducted followed by a 30 s probe trial. In all trials, animals were released facing the pool wall in a pseudo-randomized sequence. Probe trials started from the opposite quadrant (quadrant C) and lasted a maximum of 30 s. Recorded parameters included latency to reach the platform during acquisition and reversal trials, and time spent in each quadrant.

### Tissue and histological processing

#### Euthanasia and tissue processing

Ninety minutes after the final learning-phase probe trial, animals were decapitated, and brains were rapidly removed. Half of each brain hemisphere was frozen in isopentane (Sigma-Aldrich, Germany) and stored at − 40 °C until sectioning at − 20 °C in a cryostat (Leica CM1900, Germany) for cytochrome c-oxidase (CCO) histochemistry and c-Fos immunohistochemistry. Two series of 30 μm coronal sections were collected per technique. Sections were mounted on non-gelatinized slides for CCO and on gelatin and potassium dichromate-pretreated slides for c-Fos (Panreac and Sigma-Aldrich, Spain). The PFC and the hippocampus from the remaining hemisphere was isolated, frozen in liquid nitrogen, and stored at − 80 °C for western blot analysis.

#### Cytochrome c oxidase histochemistry

Sections were processed for quantitative CCO histochemistry as previously described [22]. To control staining variability across baths, tissue homogenate standards from Wistar rat brains of varying thicknesses (10, 30, 40, and 60 μm) were included in each run. Sections and standards were fixed for 5 min in 0.1 M phosphate buffer (pH 7.6) containing 10% sucrose and 25% glutaraldehyde, followed by three 5 min washes in 0.1 M phosphate buffer with 10% sucrose. They were then incubated 8 min in 0.05 M Tris buffer (pH 7.6) containing 0.275 mg/l cobalt chloride, 10% sucrose, 6 g/l Trizma base, and 0.5% DMSO. After a 5 min rinse in 0.1 M phosphate buffer, sections were incubated 1 h at 37 °C in a solution containing 0.0075% cytochrome c, 0.002% catalase, 5% sucrose, 0.25% DMSO, and 0.05% diaminobenzidine tetrahydrochloride in 0.1 M phosphate buffer. The reaction was stopped with 4% formalin for 30 min at room temperature, and slides were subsequently dehydrated in graded alcohols, cleared in xylene (Avanter, Poland), and coverslipped with Entellan (Merck, Germany).

#### c-Fos inmunohistochemistry

Sections were processed for c-Fos immunohistochemistry as previously described [23]. Sections were fixed in 4% paraformaldehyde (0.1 M, pH 7.4) for 30 min and rinsed twice in 0.01 M phosphate-buffered saline (PBS, pH 7.4). Endogenous peroxidase activity was quenched with 3% hydrogen peroxidase in PBS for 30 min, followed by two PBS washes (10 min each). Sections were then permeabilized in 1% Triton X-100 in PBS for 10 min, washed, and blocked with 3% bovine serum albumin in PBS for 30 min. Sections were incubated with rabbit polyclonal anti-c-Fos antibody (1:20,000; Synaptic Systems, Germany) in PBS containing BSA and Triton X-100 for 12 h at 4 °C in a humid chamber. After two PBS washes, sections were incubated with goat anti-rabbit biotinylated IgG (1:480; Pierce, USA) for 30 min at room temperature, followed by incubation with avidin–biotin-peroxidase complex (Vectastain ABC Ultrasensitive Elite Kit, Pierce, USA) for 30 min. Reaction was visualized using 0.05% diaminobenzidine tetrahydrochloride, 33% hydrogen peroxide, and 0.05% ammonium nickel sulfate in PBS for 3 min in darkness, then stopped with distilled water. Sections were dehydrated through graded alcohols, cleared in xylene (Avanter, Poland), and coverslipped with Entellan (Merck, Germany). Control sections omitting the primary antibody were included in all runs.

### Tissue quantification

Regions of interest (ROIs) were defined according to the Paxinos and Watson atlas (2013) [24]. For both techniques, the PFC subfields CG, PL and IL and dorsal hippocampal subfields CA1, CA3, and dentate gyrus (DG) were located at 3.24 mm and − 2.27 mm from bregma respectively. For c-Fos immunohistochemistry, hippocampal layers were further differentiated as follows: CA1 and CA3—molecular (mol), pyramidal (pyr), and polymorphic (poly) layers; DG—molecular (mol), granular (gran), and polymorphic (poly) layers.

#### Cytochrome c oxidase densitometric quantification

CCO histochemical intensity was quantified via densitometric analysis using a computer-assisted image workstation (MCID, Interfocus Imaging Ltd., Linton, UK), comprising a high-precision illuminator, digital camera, and MDCID Core 7.0 software. Mean optical density (OD) for each ROI was measured from three consecutive sections per animal, with four non-overlapping readings per section using a square-shaped dissector adjusted to region size. This resulted in 14 measurements per region per adult rat and 12 per region per aged rat. OD values were then converted to CCO activity units based on spectrophotometrically determined enzymatic activity of included standards***.***

#### c-Fos cells quantification

c-Fos positive cells were quantified by systematically sampling each ROI using counting frames superimposed on the tissue under a microscope (Leica DFC490) coupled to Leica Application Suite X software. Total magnification was 10 × for cortex and 20 × for hippocampus. Counting frame sizes were 200 × 200 µm for CG, PL, and IL and 90 × 70 µm for hippocampal subfields (CA1mol, CA1pyr, CA1poly, CA3mol, CA3pyr, CA3poly, DGmol, DGgran, DGpoly). Two frames per section per region were analyzed, yielding eight frames per region per animal. c-Fos positive nuclei were identified as homogeneously stained gray-black elements with well-defined borders. The mean c-Fos positive count across four sections was calculated for each subject and region.

### Western blot

Prefrontal and hippocampal tissues were homogenized in cold RIPA buffer (1% Triton X-100, 0.1% SDS, 0.5% sodium deoxycholate, 50 mM Tris pH 7.4, 150 mM NaCl) containing protease inhibitors (P8340, Sigma-Aldrich) at 10 µL per mg tissue. Homogenates were incubated on ice for 30 min, sonicated 20 cycles, and centrifuged (13,000 rpm, 15 min, 4 °C). Protein concentration was determined using the DC Protein Assay kit (BioRad), and 10 µg of protein per sample was mixed with SDS loading buffer and boiled at 95 °C for 5 min.

Samples were separated by SDS-PAGE using MOPS (NP0001, Invitrogen) or MES buffer (NP0002, Invitrogen) and transferred to nitrocellulose membranes (iBlot2, 7 min, 20 V). Membranes were blocked with 3% milk in TBS and incubated overnight with primary antibodies diluted in TBS containing 0.2% Tween 20 and 1% milk. The following antibodies were used: NeuN (ab104224, Abcam), GFAP (173008, Synaptic System), Iba-1 (ab178846, Abcam), Synapsin-I (ab64581, Abcam), PSD95 (ab13552, Abcam), ERK (EM1221, ELK Biotechnology), p-ErK (4370, Cell Signaling Technology), NF-κB (EM1111, ELK Biotechnology), TNF-α (ab183218, Abcam), p38 (ES4838, ELK Biotechnology), p53 (EM1052, ELK Biotechnology), BCL-2 (EM1041, ELK Biotechnology), IL-1β (12242, Cell Signaling Technology), IL-6 (ES4078, ELK Biotechnology) and IL-10 (ab9969, Abcam). Protein levels were normalized with GAPDH (G8795, SIGMA) for each sample.

Detection employed fluorophore-conjugated secondary antibodies (IRDye® 800CW Goat anti-Mouse 926–32210, IRDye® 680RD Goat anti-Mouse IgG 926–68070, VRDye™ 549 Goat anti-Mouse IgG 926–54010, IRDye ® 800CW Goat anti-Rabbit IgG 926–32211, IRDye® 680RD Goat anti-Rabbit IgG 926–68071, IRDye® 680RD Donkey anti-Goat IgG 926–68074 from LICOR) diluted at 1:20.000 or 1:10.000, following the recommendations manufacturer. Specific binding was visualized using an Odyssey M scanner (LICOR), and protein levels were quantified with Empiria Studio 3 software.

### Statistical analysis

Data were assessed for normality (Shapiro–Wilk test) and homogeneity of variance (Brown-Forsythe test) to determine the appropriate use of parametric or non-parametric tests. Behavioral data from MWM probe trials, expressed as time spent in each quadrant, were analyzed using two-way ANOVA or Kruskal–Wallis tests, with Holm-Sidak or Dunn’s post-hoc comparisons, respectively. Escape latencies were evaluated using repeated-measures ANOVA within each group, followed by the same post-hoc analyses.

CCO and c-Fos measurements were compared between groups by one-way ANOVA with Holm-Sidak post-hoc testing. Protein quantification was performed by densitometric analysis of immunoblots, and group comparisons were assessed using one-way ANOVA followed by Tukey’s multiple comparisons test. All statistical analyses and graphical representations were performed using GraphPad Prism 10, with significance set at *P* < 0.05.

## Results

### PBM frequencies have differential effects on spatial memory performance

**Fig. 1:**
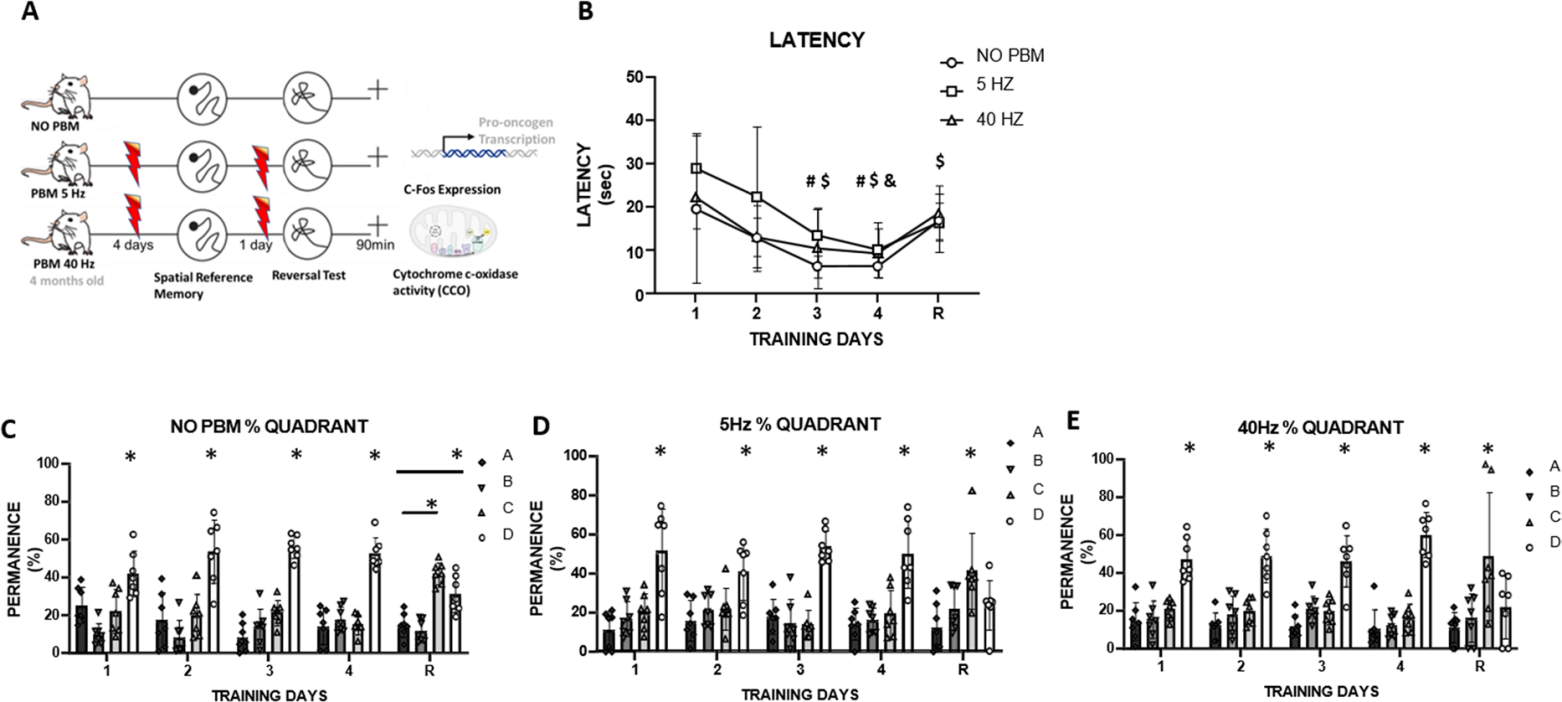
PBM frequencies have differential effects on spatial memory performance. **a** Experimental protocol: 7 male adult rats per group were subjected to the presence or absence of PBM under non-activated and activated networks by the spatial reference memory. **b** Latency to find the platform during acquisition and during the reversal trial. Differences in memory performances of 40 Hz,5 Hz and control rats during the training days were found. The NO PBM group, the latency results showed significant differences between day 1 and days 3 and 4 (#p < 0.033). In the PBM 5 Hz group, significant differences in latencies were observed between day 1 and days 3, 4 and R ($p < 0.05). For the PBM 40 Hz group, significant differences in latencies were found between days 1 and 4 (&*p* < 0.039). **c** Time spent in the target quadrant (D) during the training days, and in the reversal test (quadrant C) for the NO PBM group. Time spent in the target quadrant was higher during the training days, whereas in the reversal test, a greater amount of time was shared between the new target quadrant (C) and the previously reinforced quadrant (D) compared to the other quadrants (A and B). (Tukey’s test: *p < 0.05). **d** Time spent in the target quadrant (D) was higher during the training days, and in the reversal test (C) for the 5 Hz group. (Tukey’s test: *p < 0.05). **e** Time spent in the target quadrant (D) was higher during the training days, and in the reversal test (C) for the 40 Hz group. (Tukey’s test: *p < 0.05) Each dot represents an individual animal; bars indicate mean ± SEM

We first aimed to assess spatial memory performance in young adults. Differences were observed in the latencies along the training days (F_(4,90)_ = 10.82; *p* < 0.0001) (Fig. 1B). Specifically, NO PBM group showed differences in day 1 compared to day 3 and 4, the 5 Hz group revealed differences in day 1 compared to the rest of the days, and the40 Hz group only showed a difference between day 1 and 4 (Fig. 1B). The percentage of time spent by the subjects during the probe tests across the training days revealed significant differences in the target position (quadrant D from day 1 to day 4 and quadrant C on day 5:R). On day 5:R, PBM-treated rats spent more time in the reinforced quadrant (C) compared to the NO PBM group (F_(3,120)_ = 95.52; *p* < 0.0001), with the effect being slightly greater in the 40 Hz group (F_(3,120)_ = 48.65; *p* < 0.0001) than in the 5 Hz group (F_(3,120)_ = 41.65; *p* < 0.0001) (Fig. 1C–E).

### The effect of PBM on metabolic activity and pro-oncogen expression in young animals

**Fig. 2:**
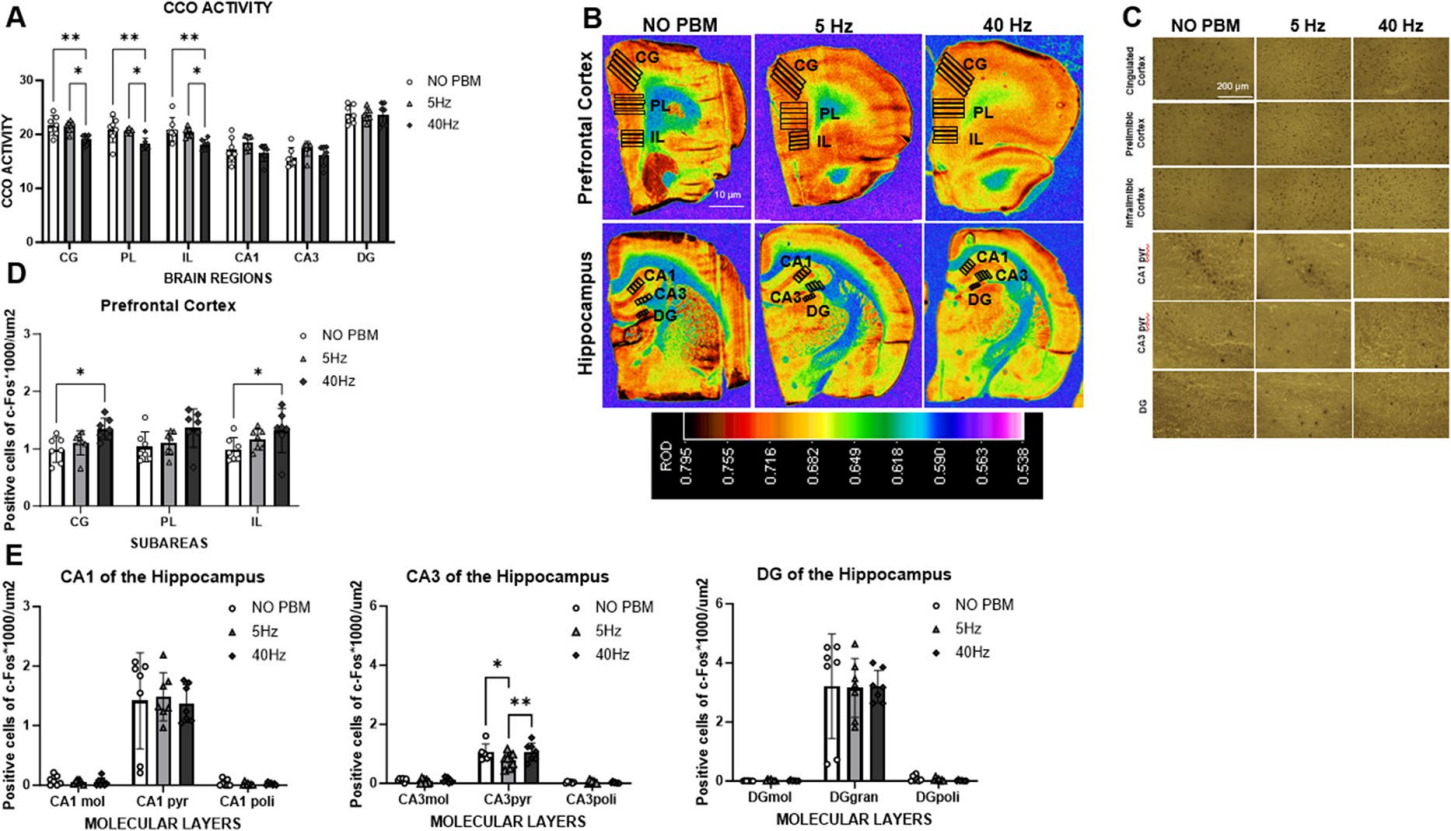
The effect of PBM is dependent on the activation state of the brain network. **a** CCO activity in the NO PBM, 5 Hz and 40 Hz groups across the PFC and hippocampus. **b** CCO histochemical intensity illustration showing different activity among experimental groups where red/brown indicates the maximum activity and pink the lowest. Bar scale 10 µm. **c** c-Fos immunoreactivity illustration showing different pro-oncogen expression among experimental groups. Bar scale 200 µm. **d** c-Fos immunoreactivity in the three experimental groups across the prefrontal cortices. **e** c-Fos immunoreactivity in the three experimental groups across the hippocampal layers. Each dot represents an individual animal; bars indicate mean ± SEM. Statistical significance was determined using one-way ANOVA followed by Tukey’s tests (*p < 0.05, **p < 0.01)

We assessed and compared the metabolic effects of different PBM frequencies in a functionally active neural brain network during the execution of the spatial reversal memory test. Differences were found between experimental groups (F_(2,108)_ = 14.67; *p* < 0.0001). The results demonstrated (Fig. 2A) an activation of a network that resulted in a decrease in CCO activity in the 40 Hz group compared with controls (CG: *p* = 0.0073; PL: *p* = 0.0052; IL: *p* = 0.0027). Also, a decrease was observed in the 40 Hz group, compared with 5 Hz (CG: *p* = 0.0114; PL: *p* = 0.0111; IL: *p* = 0.0247). The number of c-Fos positive nuclei reported differences (F_(2,54)_ = 9.582; *p* = 0.0003) between two groups (NO PBM and 40 Hz) in the PFC areas such as CG (*p* = 0.0271) and IL (p = 0.0491; Fig. 2D). Also, differences in the layers of the hippocampus were found between experimental groups (F_(2,54)_ = 201.7; *p* < 0.0001) in the pyramidal layer of CA3 of the hippocampus, where c-Fos expression was decreased in the 5 Hz group compared to NO PBM (*p* = 0.0158) and 40 Hz (*p* = 0.0067; Fig. 2E).

### Changes in frequency patterns in PBM produces changes in gliogenesis and synaptogenesis

**Fig. 3:**
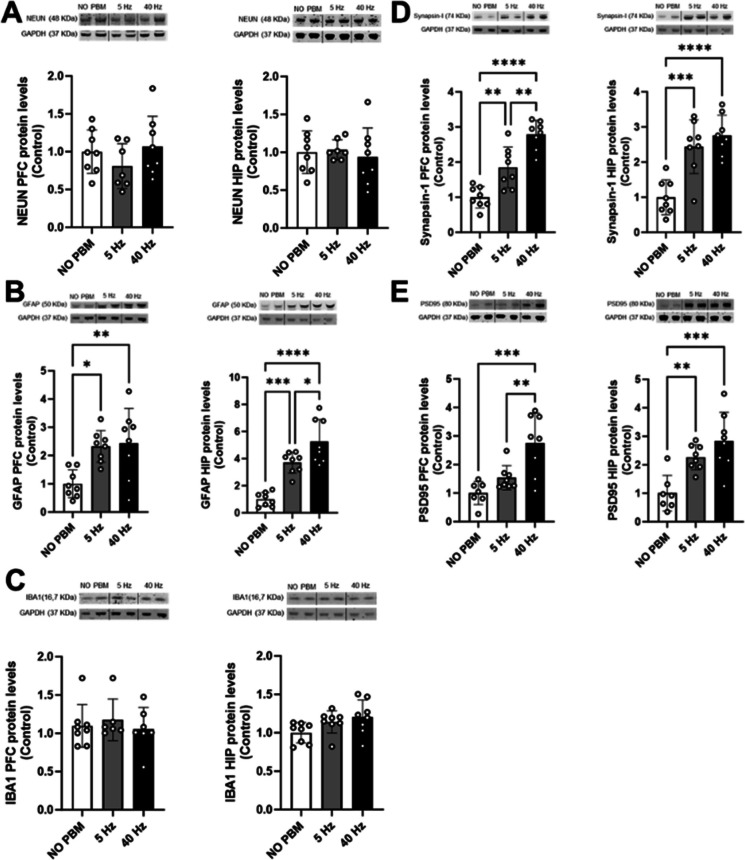
PBM frequency-dependent modulation of synaptogenesis and gliogenesis. Representative western blots (top) and quantification (bottom) of protein expression in PFC and hippocampal samples from rats treated with 5 Hz or 40 Hz PBM or without PBM. Panels show levels of **a** NeuN, **b** GFAP, **c** Iba-1, **d** Synapsin-I, **e** PSD-95. Each dot represents an individual animal; bars indicate mean ± SEM. Statistical significance was determined using one-way ANOVA followed by post hoc tests (*p < 0.05, **p < 0.01, ***p < 0.001, ****p < 0.0001)

Immunoblots were performed to quantify neuron and glial cells expression levels as well as synaptogenesis general markers. Whereas no changes were found in neuronal marker (NeuN) between conditions in any brain region such as prefrontal cortex (F _(2, 20)_ = 1.184; *p* = 0.3267) and hippocampus (F _(2, 20)_ = 0.2057; *p* = 0.8158), an increase in astroglia marker (GFAP) in prefrontal cortex (F_(2,21)_ = 7.434; *p* = 0.0036) and hippocampus (F_(2,21)_ = 31.47; *p* < 0.0001) was found when applied 5 Hz (PFC: *p* = 0.0115; HIP: *p* = 0.0002) and 40 Hz (PFC: *p* = 0.063; HIP: *p* < 0.0001) versus the group without PBM. Also, hippocampal expression increased when applied 40 Hz in comparison to 5 Hz (*p* = 0.0248). Contrary, no changes in the microglia (Iba-1) expression were observed between groups in any brain region such as prefrontal cortex (F _(2, 20)_ = 0.3116; *p* = 0.3267) and hippocampus (F _(2, 21)_ = 2.986; *p* = 0.0722) (Fig. 3A-C).

Regarding synaptic proteins, an increase in Synapsin-I in prefrontal cortex (F_(2,21)_ = 32.55; *p* < 0.0001) and hippocampus (F_(2,21)_ = 18.18; *p* < 0.0001) was observed when applied 5 Hz (PFC: *p* = 0.026; HIP: *p* = 0.0004) and 40 Hz (PFC: *p* < 0.0001; HIP: *p* < 0.0001) versus the group without PBM. Also, prefrontal expression increased when applied 40 Hz in comparison to 5 Hz (*p* = 0.0011). Surprisingly, differences in PSD-95 expression were observed across frequencies in prefrontal cortex (F_(2,20)_ = 12.18; *p* = 0.0003) and hippocampus (F_(2,20)_ = 12.22; p = 0.0003). Specifically, the 40 Hz frequency increased PSD-95 expression in more anterior regions such as the PFC compared to the 5 Hz frequency (*p* = 0.0073) and the NO PBM group (*p* = 0.0003). In contrast, both the 40 Hz (*p* = 0.0003) and 5 Hz (*p* = 0.0086) frequencies elevated the expression of this postsynaptic protein in the hippocampus (Fig. 3D-E).

### PBM shapes brain inflammation in a frequency- and region-specific manner

**Fig. 4:**
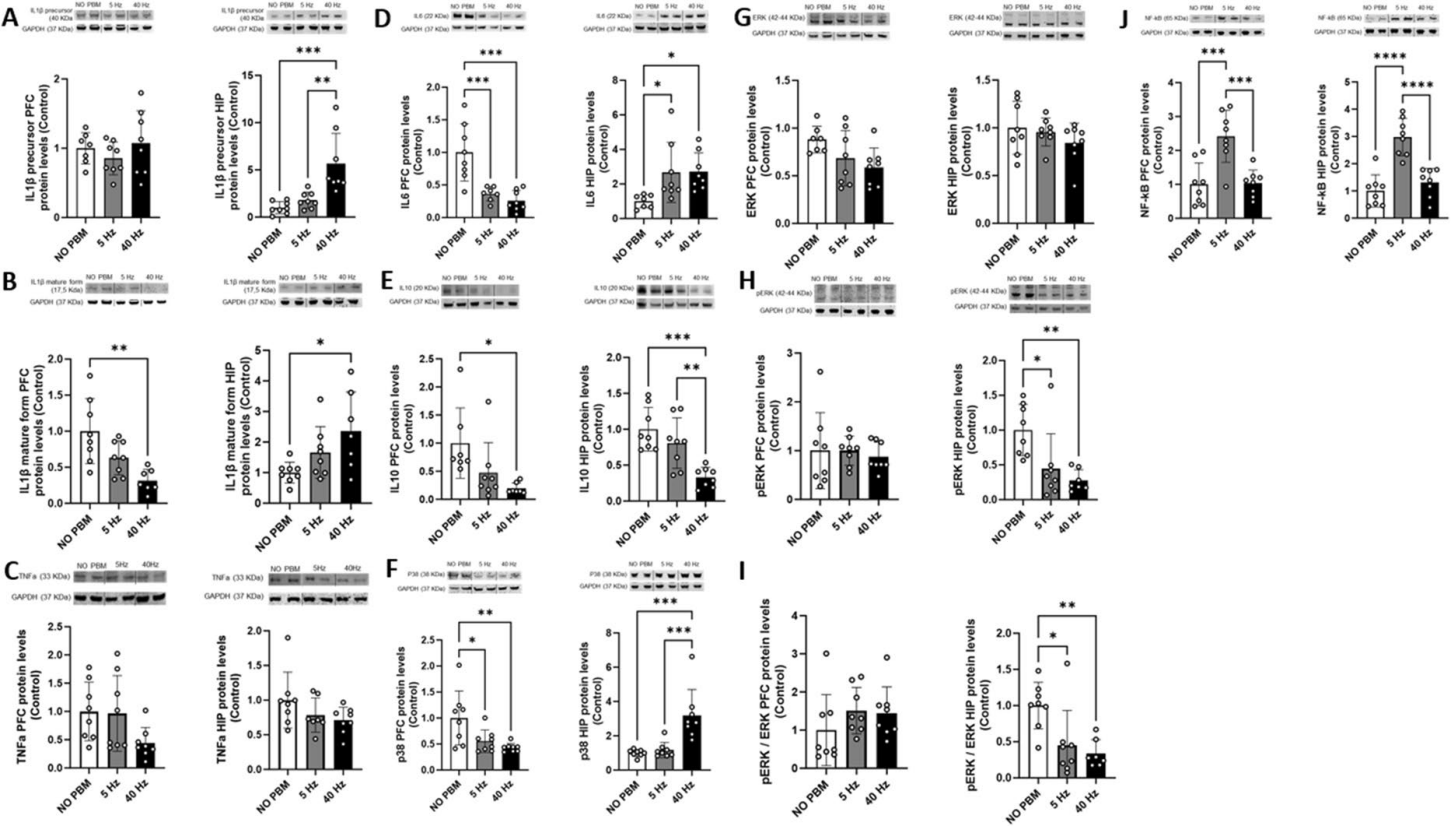
PBM frequency-dependent modulation of neuroinflammation. Representative western blots (top) and quantification (bottom) of protein expression in PFC and hippocampal samples from rats treated with 5 Hz or 40 Hz PBM or without PBM. Panels show levels of **a** IL-1 β precursor, **b** IL-1 β mature form, **c** TNF-α, **d** IL-6, **e** IL-10, **f** p38 MAPK, **g** ERK,** h** pERK, **i** ratio pERK/ERK, **j** NF-κB. Each dot represents an individual animal; bars indicate mean ± SEM. Statistical significance was determined using one-way ANOVA followed by post hoc tests (*p < 0.05, **p < 0.01, ***p < 0.001)

At the site of application, the PFC showed decreased expression of IL-1β mature form (F_(2,20)_ = 9.025; *p* = 0.0016) at 40 Hz (*p* = 0.0011) and IL-10 (F_(2,19)_ = 5.046; *p* = 0.0175) at 40 Hz (*p* = 0.0147) and a reduction in expression was found in IL-6 (F_(2,20)_ = 15.93; *p* < 0.0001) when applying both 5 and 40 Hz (*p* = 0.0009; *p* = 0.0001 respectively). No changes were observed at the application site in the expression of the precursor form of IL-1β (F_(2,20)_ = 0.8768; *p* = 0.4315) and TNF-α (F_(2,21)_ = 2.963; *p* = 0.0736) (Fig. 4A-E).

Regarding the hippocampus, we have observed an increase in hippocampal expression at 40 Hz in the mature form of IL-1β (F_(2,20)_ = 4.410; *p* = 0.0259) when compared to NO PBM (*p* = 0.0198). Also, changes were observed in the precursor form of IL-1β in the hippocampus (F_(2,21)_ = 13.00; *p* = 0.0002), where its expression was significantly increased at 40 Hz compared to the control (*p* = 0.0003) and 5 Hz (*p* = 0.0020). Similar increase in protein expression was observed for IL-6 (F_(2,20)_ = 4.573; *p* = 0.0232) at 5 Hz (*p* = 0.0420) and 40 Hz (*p* = 0.0368). Contrary to this, decreases in hippocampal levels of IL-10 (F_(2,21)_ = 12.09; *p* = 0.0003) were found at 40 Hz compared to 5 Hz (*p* = 0.0074) and NO PBM (*p* = 0.0003; Fig. 4A-E). No differences were found in TNF-α (F_(2,20)_ = 2.072; *p* = 0.1522).

A similar pattern of expression was observed in p38 MAPK. Specifically, an increase was detected in the hippocampus (F_(2,21)_ = 14.10; *p* = 0.0001) with the application of 40 Hz compared to both the control (*p* = 0.0003) and 5 Hz (*p* = 0.0007) conditions. In contrast, both frequencies (5 Hz and 40 Hz) led to a decrease in p38 MAPK expression in the PFC (F_(2,21)_ = 6.620; p = 0.0059) when compared to the control (p = 0.0337; *p* = 0.0064, respectively; Fig. 4F).

It is important to note the hippocampal decrease of pERK (F_(2,20)_ = 7.683; *p* = 0.0033) when applying both frequencies (5 and 40 Hz) when compared to control (*p* = 0.0216; *p* = 0.0039) and their ratio (pERK/ERK) (F_(2,20)_ = 7.558; *p* = 0.0036) when compared to control (*p* = 0.0159; *p* = 0.0052; Fig. 4G-I).

5 Hz increased NF-κB expression (F_(2,21)_ = 13.87; *p* = 0.0001) compared to control and 40 Hz at PFC (*p* = 0.0004; *p* = 0.0005) and hippocampus (F_(2,21)_ = 25.13; p < 0.0001) in both comparisons (*p* < 0.0001); Fig. 4J).

### PBM decreases apoptotic pathways

**Fig. 5:**
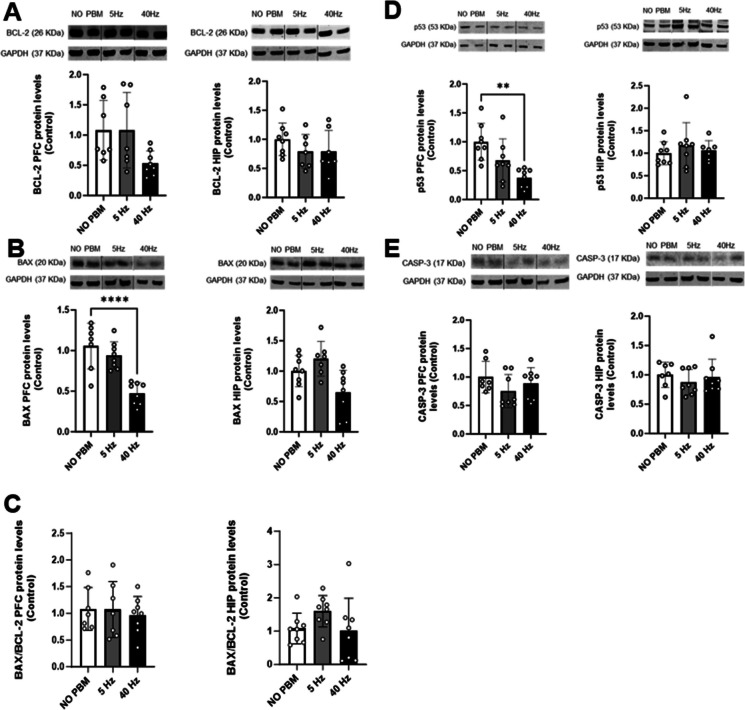
Selective reduction of BAX and p53 by 40 Hz PBM in the PFC. Representative western blots and quantification of apoptosis-related proteins in PFC and hippocampus from rats treated with 5 Hz PBM, 40 Hz PBM, or no PBM. Panels show levels of **a** BCL-2, **b** BAX, **c** BAX/BCL-2 ratio, **d** p53, **e** CASP-3. 40 Hz PBM selectively reduced BAX and p53 expression in the PFC, with no changes in BCL-2, CASP-3, or BAX/BCL-2 ratio in either region. Data are shown as mean ± SEM, each dot represents one animal; one-way ANOVA with post hoc tests (**p < 0.01, ****p < 0.0001)

In regards of the mitochondrial apoptosis, a reduction in BAX expression was found in the PFC (F_(2,20)_ = 18.36; *p* < 0.0001) at 40 Hz (*p* < 0.001; Fig. 5B) which was not accompanied by differences in the hippocampus (F_(2,20)_ = 6.312; *p* = 0.0075).

In terms of p53 expression, we observed a decrease in expression at the PFC (F_(2,21)_ = 8.800; *p* = 0.0017) when applying 40 Hz in comparison to NO PBM (*p* = 0.0012; Fig. 5D). Finally, no differences were found in CASP3, BCL-2 or the ratio BAX/BCL-2, apoptotic and anti-apoptotic markers (Fig. 5A, C and E), in prefrontal (CASP3: F_(2,20)_ = 1.460; *p* = 0.2560; BCL-2: F_(2,19)_ = 3.529; p = 0.05; BAX/BCL-2: F_(2,19)_ = 0.1744; *p* = 0.8413) neither hippocampus (CASP3: F_(2,20)_ = 0.4887; *p* = 0.6205; BCL-2: F_(2,19)_ = 0.08351; *p* = 0.9202; BAX/BCL-2: F_(2,19)_ = 1.558; *p* = 0.2363), indicating that apoptosis is not fully engaged.

### Therapeutic effects of the PBM-40 Hz on recovery cognitive decline

**Fig. 6:**
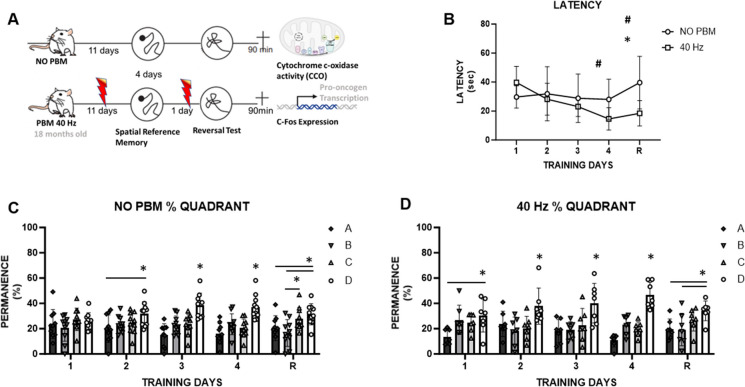
PBM accelerates spatial learning and improves cognitive flexibility in aged rats. **a** Experimental protocol: 7 male 18 months aged rats per group were subjected to the presence or absence of PBM under activated networks by the spatial reference memory. They were trained for 4 days in the MWM, and a reversal test was performed on the next day (day 5). 90 min after the learning task the animals were sacrificed, and CCO and c-Fos expression were measured in the brain regions involved. **b** Latency to find the platform during acquisition (D1-D4) and during the reversal trial (R). Differences in memory performances of PBM 18 M compared to those of the same age without PBM during the reversal trial were found. For the NO PBM group, the latency results showed no significant differences across the training days. In the 40 Hz group, significant differences in latencies were observed between day 1 and the last two training days. **c** Time spent in reinforced and non-reinforced quadrants during the learning phase (D1-D4) and the reversal test (R) showed distinct patterns between groups. Each dot represents an individual animal; bars indicate mean ± SEM. Statistical significance was determined using repeated measures ANOVA followed by Tukey’s tests (*p < 0.05 between groups, #*p* < 0.05 between days)

Regarding the spatial memory performance in aged animals, significant differences were observed between experimental groups in the latencies along training days (F_(1,60)_ = 4.999; *p* = 0.0291; Fig. 6B). Differences in latencies were observed between the later days (day 5 and 4 compared to day 1) which were not observed in the NO PBM group.

The percentage of time spent by the subjects during the probe tests across the training days revealed significant differences in the target position (F_(3,36)_ = 16.49; *p* < 0.0001) (quadrant D from day 1 to day 4 and quadrant C on day 5, R). Specifically, significant differences between the others quadrants and the reinforced quadrant occurred on day 3 (D vs A: *p* < 0.001; D vs B: *p* = 0.0035; D vs C: *p* = 0.0062) and 4 (D vs A: *p* = 0.0003; D vs C: *p* = 0.0023; D vs B: *p* = 0.0159), whereas on day 5 differences were only found between quadrant D and B (*p* = 0.0140). Similarly, the PBM-treated group exhibited learning (F_(3,24)_ = 24.35; *p* < 0.0001) from day 3 showing the highest preference for the reinforced quadrant over all others (D vs A: *p* = 0.0225; D vs B: *p* = 0.0188; D vs C: *p* = 0.0169) and day 4 (D vs A: *p* < 0.0001; D vs B: *p* = 0.0007; D vs C: *p* = 0.0001), whereas on day 5 differences were only found between quadrant D and A (*p* = 0.0013). When quadrant C was reinforced on the final day, animals from both groups continued to show perseveration toward the previously reinforced quadrant.

### The effect of PBM on mitochondrial activity and proto-oncogene expression in aged animals

**Fig. 7:**
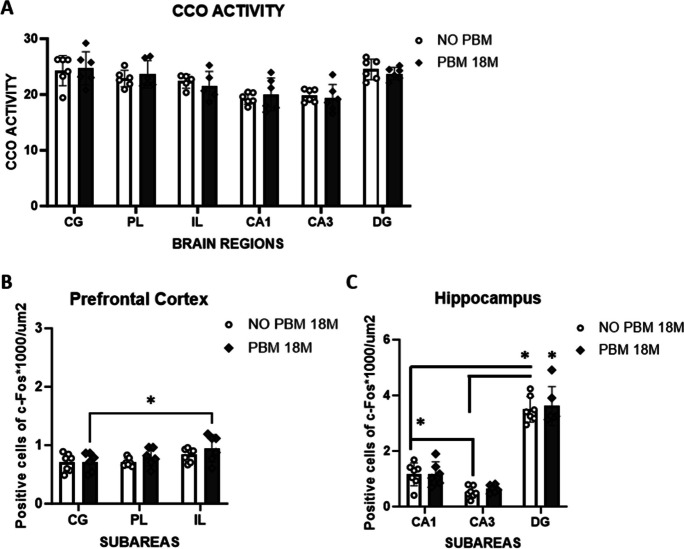
PBM modulates c-Fos expression but not mitochondrial CCO activity in aged brain regions **a** No significant differences in CCO activity were observed between groups **b** c-Fos activity in the different subareas of the PFC **c** Significant differences were observed among hippocampal subregions, and the magnitude and distribution of these differences varied as a function of PBM application. Each dot represents an individual animal; bars indicate mean ± SEM. Statistical significance was determined using one-way ANOVA followed by Tukey´s tests (*p < 0.05, ****p < 0.0001)

Next, we examined whether the cognitive improvements induced by PBM in aged animals were linked to mitochondrial activity and proto-oncogene expression by assessing CCO activity and c-Fos expression. CCO activity did not differ between groups (F_(1,58)_ = 0.0044; *p* = 0.9475; Fig. 7A). In contrast, PBM with 40 Hz increased c-Fos expression (F_(2,33)_ = 5.013; *p* = 0.0126), with greater activation in the IL relative to the CG (*p* = 0.03; Fig. 7B). Within the hippocampus differences were found (F_(2,33)_ = 176.1; *p* < 0.0001), the NO PBM group showed significant differences across all subregions (CA1 vs CA3: *p* = 0.0231; CA1 vs DG: p < 0.0001; CA3 vs DG: p < 0.0001), whereas in PBM-treated animals differences were detected between CA1 and DG (*p* < 0.0001) and CA3 vs DG (*p* < 0.0001; Fig. 7C).

### Study of the clinical significance of PBM-40 Hz in aging

**Fig. 8:**
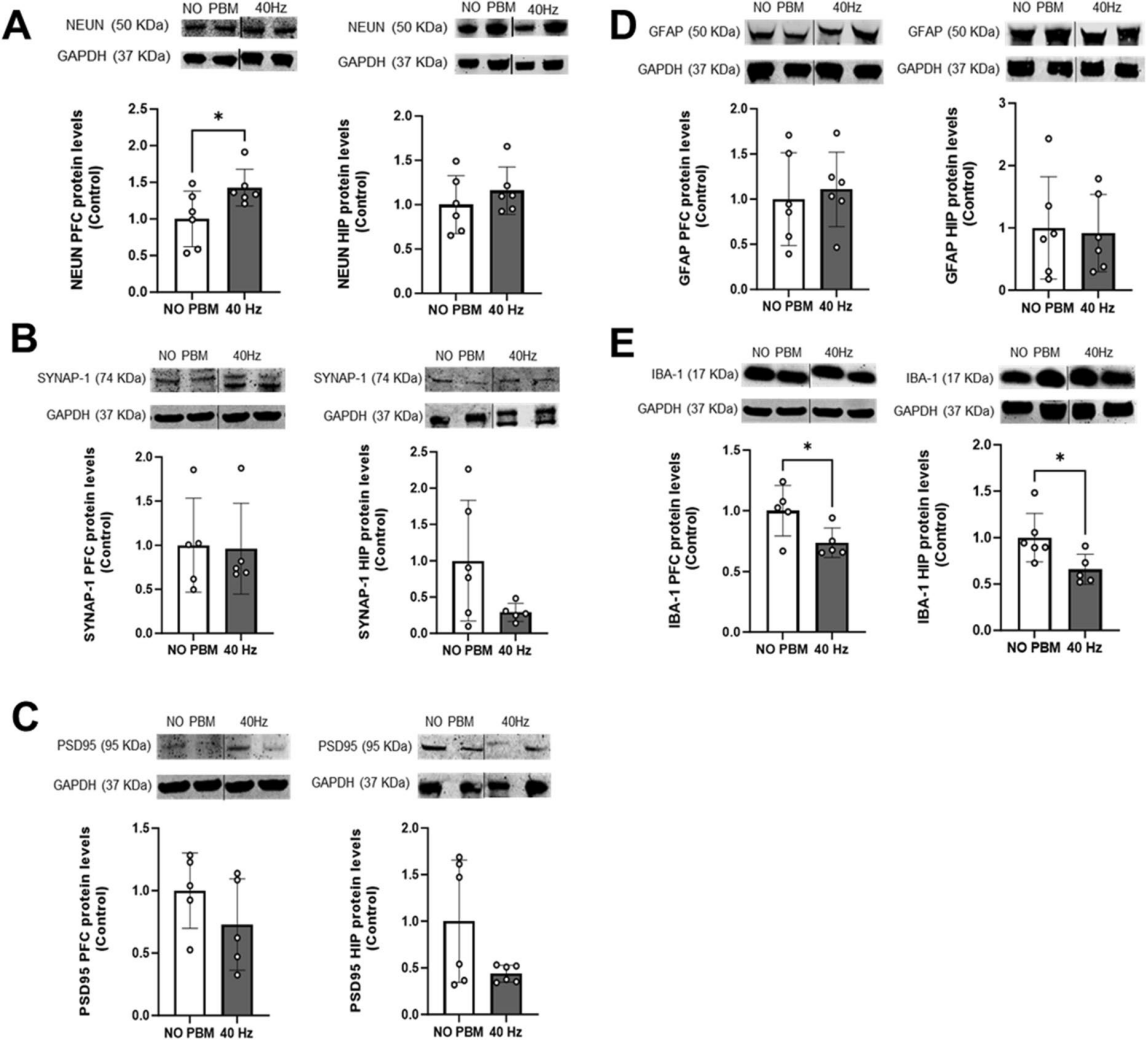
PBM prevents neuronal loss and reduces inflammation without altering synaptic or glial markers. Representative western blots and quantification of inflammatory and neuroplasticity proteins in PFC and hippocampus from aged rats treated with 40 Hz PBM, or no PBM. Panels show levels of **a** NeuN, **b** Synapsin-I, **c** PSD-95 **d** GFAP **e** Iba-1. PBM in the PFC prevents neuronal loss, as indicated by sustained NeuN expression, and reduces inflammation, reflected by decreased Iba1 levels in both the PFC and hippocampus of 18-month-old rats, without affecting PSD-95, Synapsin-I, or GFAP expression. Data are shown as mean ± SEM, each dot represents one animal; one-way ANOVA with Tukey’s tests (*p < 0.05)

Immunoblots were performed to quantify neurons or glial cell levels as well as general synaptogenesis markers in aged animals. We have found an increase in NeuN when applying PBM in the PFC (t_10_ = 2.298; *p* = 0.0444), which was accompanied by a decrease in Iba-1 expression at the PFC (t_8_ = 2.445; *p* = 0.0402) and the hippocampus (t_9_ = 2.541; *p* = 0.0317). No alterations in neuroplasticity markers such as PSD-95 (t_8_ = 1.281; *p* = 0.2361; t_9_ = 1.878; *p* = 0.1008, respectively) or Synapsin-I (t_8_ = 0.1219; *p* = 0.9060; t_9_ = 1.878; *p* = 0.0931, respectively), neither were detected in GFAP in the PFC (t_10_ = 0.3993; *p* = 0.6981) or hippocampus (t_10_ = 0.1989; *p* = 0.8463) at 18 months (Fig. 8A-E).

**Fig. 9:**
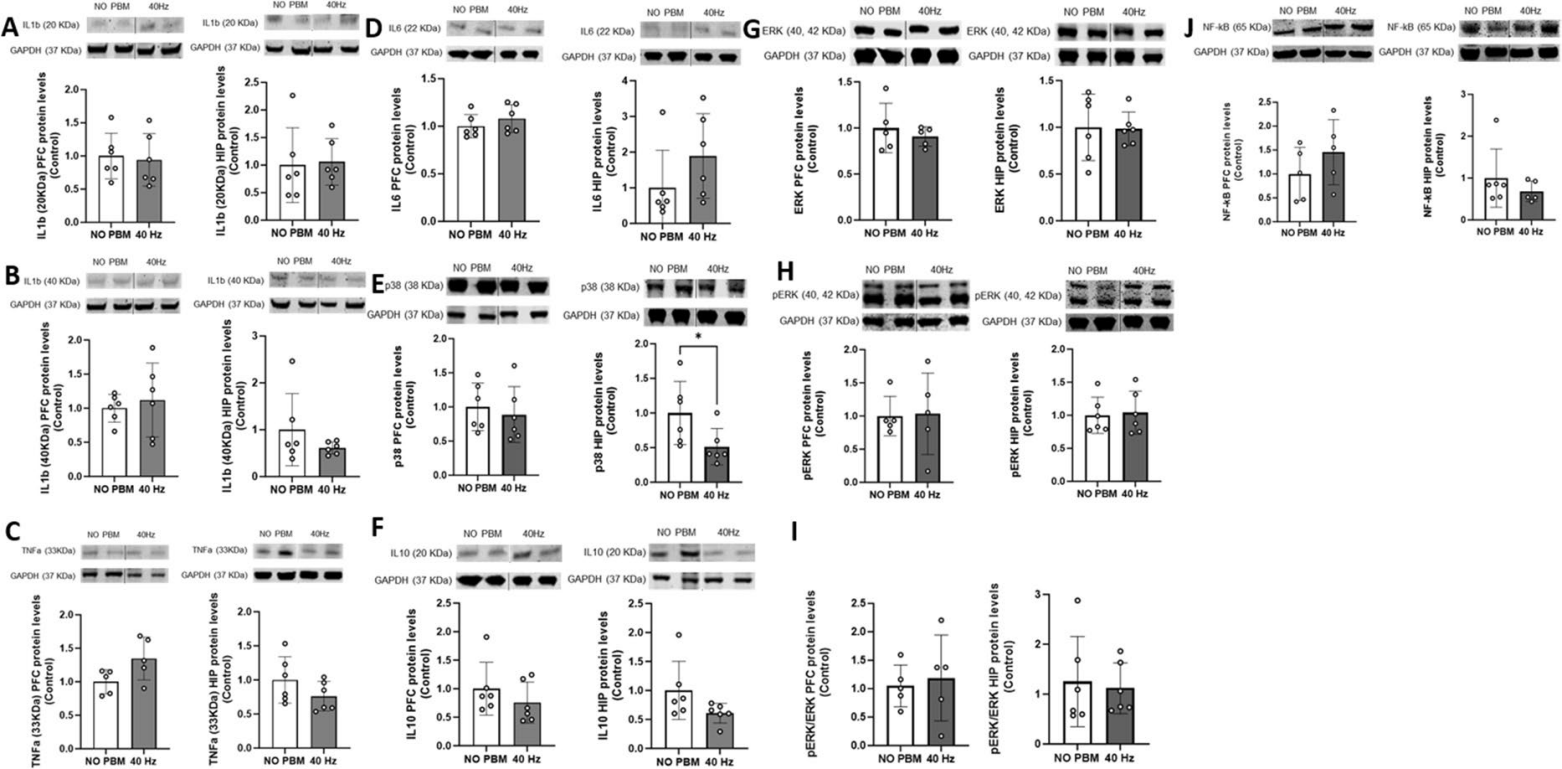
PBM reduces hippocampal p38 MAPK expression in aged rats but does not affect other inflammatory signaling markers. **a-b** IL 1b **c** TNF-α **d** IL-6 **e** p38 **f** IL-10 g ERK **h** pERK **i** pERK/ERK **j** NF- κB reduced p38 MAPK expression in the hippocampus. Data are shown as mean ± SEM, each dot represents one animal; one-way ANOVA with post hoc tests (*p < 0.05)

Similarly, no significant differences were found in any inflammatory markers, including IL-1β (both its precursor (PFC: t_10_ = 0.5065; *p* = 0.7911; HIP: t_10_ = 1.215; *p* = 0.2524) and mature form (PFC: t_10_ = 0.2721; *p* = 0.7911; HIP: t_10_ = 0.1798; *p* = 0.8609), TNF-α (PFC: t_8_ = 2.120; *p* = 0.0669; HIP: t_10_ = 1.450; *p* = 0.0.1777), IL-6, (PFC: t_10_ = 1.039; *p* = 0.3235; HIP: t_10_ = 1.3768; *p* = 0.1988), IL-10 (PFC: t_10_ = 1.014; *p* = 0.3343; HIP: t_10_ = 1.810; *p* = 0.1004), NF-κB (PFC: t_8_ = 1.154; *p* = 0.2818; HIP: t_9_ = 0.9559; *p* = 0.3641), ERK (PFC: t_8_ = 0.7108; p = 0.4974; HIP: t_10_ = 0.09778; *p* = 0.9240), or its phosphorylated form, pERK (PFC: t_8_ = 0.1087; *p* = 0.9161; HIP: t_10_ = 0.2261; *p* = 0.8257), as well as their ratio (PFC: t_8_ = 0.3680; *p* = 0.7224; HIP: t_10_ = 0.3116; *p* = 0. 7618). The only significant differences were observed in the hippocampus, where p38 MAPK expression was reduced (t_10_ = 2.261; *p* = 0.0473; Fig. 9A-J).

**Fig. 10:**
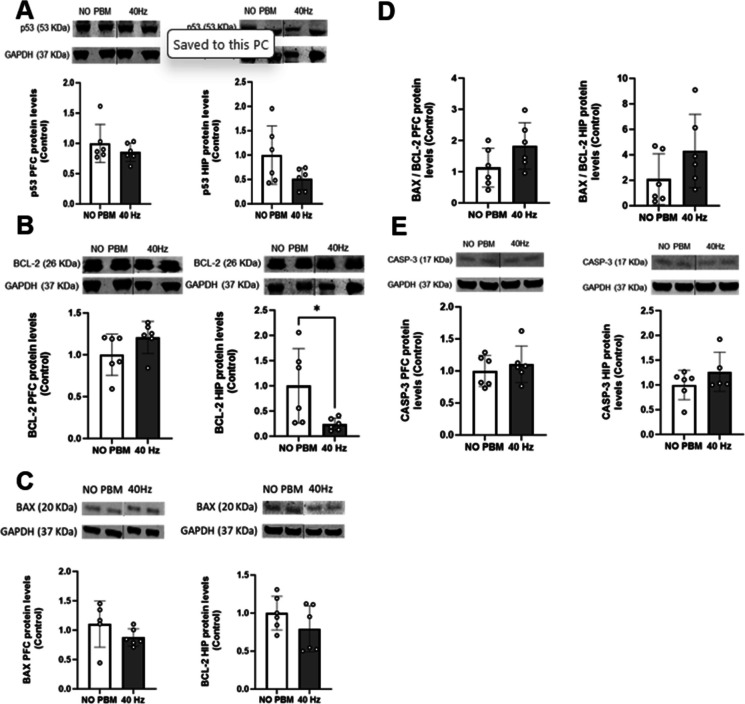
PBM downregulates hippocampal BCL-2 expression in aged rats while leaving other apoptotic markers unaffected. **a** p53 **b** BCL-2 **c** BAX **d** BAX/BCL-2** e** CASP-3. Data are shown as mean ± SEM, each dot represents one animal; one-way ANOVA with post hoc tests (*p < 0.05)

Finally, a reduction in apoptotic activity was indicated by the lower expression of BCL-2 in the hippocampus (t_10_ = 2.495; *p* = 0.0317) whereas no other changes were observed in the apoptotic markers as p53 (PFC: t_10_ = 1.018; *p* = 0.3327; HIP: t_9_ = 1.867; *p* = 0. 0914), CASP-3 (PFC: t_10_ = 0.6863; *p* = 0.5081; HIP: t_9_ = 1.271; *p* = 0. 2357), BAX (PFC: t_9_ = 1.318; *p* = 0.2200; HIP: t_10_ = 1.350; *p* = 0.2069) or its ratio BAX/BCL-2 (PFC: t_10_ = 1.768; *p* = 0.1075; HIP: t_10_ = 1.554; *p* = 0. 1512) (Fig. 10A-E).

## Discussion

In this study, we investigated the impact of frequency-specific transcranial PBM applied to the prefrontal cortex of adult rats and the effect of PBM at 40 Hz applied to the prefrontal cortex of aged rats. Both 5 Hz and 40 Hz stimulation improved cognitive performance in young adults, although 40 Hz induced more pronounced and immediate effects, particularly enhancing prefrontal activation and modulating prefrontal c-Fos expression. These behavioral gains were accompanied by frequency-dependent regulation of neuroinflammatory pathways—including differential modulation of IL-6, IL-10, and NF-κB—as well as increased expression of the synaptic proteins Synapsin-1 and PSD-95, indicating enhanced synaptic plasticity. Importantly, when applied to aged animals, 40 Hz PBM accelerated learning, attenuated hippocampal subregional differences, reduced microglial activation, and preserved neuronal integrity.

NIR light delivered via PBM can modulate neural activity and improve cognitive function [25]. PBM has been shown to enhance cognition in adults with traumatic brain injury [26, 27] and to mitigate age-related cognitive decline [28], dementia [29], and neurodegeneration [30]. PBM has been investigated in humans using non-invasive devices with several clinical studies reporting cognitive benefits. For example, a randomized, double blind, placebo-controlled trial found that PBM significantly improved cognitive performance and increased BDNF levels in older adults with mild cognitive impairment, compared to sham stimulation [31]. In another proof-of-concept study, PBM delivered via an 810 nm LED headset improved attention, memory and executive functioning in adults with a history of repetitive head acceleration events [32]. Moreover, systematic reviews of human PBM studies indicate that a majority report positive effects on cognitive outcomes across healthy and clinical populations, highlighting the translational relevance of light-based neuromodulation [33].

However, the impact of PBM stimulation frequency on active neural networks during cognitive flexibility tasks remains largely unexplored, particularly across age groups.

Previous animal studies report that PBM enhances spatial memory in aging [34], mainly through chronic irradiation with red or NIR light (660–1072 nm) [35–37]. Many of these studies, however, lacked systematic variation of stimulation frequency or focused on passive learning paradigms.

Here, we demonstrate frequency-specific effects of PBM under task-engaged conditions in young adult rats and that PBM treatment at 40 Hz supports the consolidation and retrieval of spatial memory processes that are typically vulnerable to age-related decline. Research investigating the effects of PBM parameters on behavior has consistently demonstrated positive outcomes, particularly with PW stimulation within the 40–200 mW range. Studies have shown that 40 Hz pulsed stimulation at 40 mW significantly enhances behavioral performance, while higher intensities such as 200 mW, produce stronger cognitive benefits [38]. Additionally, PBM using an 808 nm wavelength, whether in continuous mode or at 40 Hz pulsed stimulation, has shown comparable advantages, reinforcing the effectiveness of this frequency [39]. Moreover, evidence suggests that higher doses (32 J/cm^2^) are more effective than lower ones (16 J/cm^2^) in improving cognitive function [40]. Given these findings, we selected a PBM treatment tailored for aging, aligning with parameters previously shown to enhance learning and memory. To replicate the successful behavioral outcomes observed in prior research, we applied PBM for a prolonged duration, ensuring consistency with the reported effective parameters (45.8 mW/cm^2^ and 33 J/cm^2^).

To be sure to detect the effect of each frequency on consolidated spatial information, we analysed the time spent to reach the platform during 60 s. The impact of PBM on memory was tested 1 day later during a reversal test. In young animals, both 5 Hz and 40 Hz improved cognitive flexibility during reversal tests, reflecting enhanced adaptability to a changed target location, a behavioral marker of prefrontal-hippocampal network function. Regarding the aged animals, 40 Hz stimulation accelerated escape latency reduction, indicative of faster learning. These findings suggest that PBM stimulation enhances cognitive flexibility, demonstrating more effective adaptation to the changed target location compared to the control group. They also provide the first evidence that temporally patterned PBM can modulate not only memory but also cognitive flexibility, revealing mechanistic insight into modulation of neural plasticity in the aging brain.

To explore the mechanisms underlying these frequency-dependent behavioral improvements, we focused on the cellular and molecular effects of PBM, including photon absorption by CCO [41, 42] a key enzyme of the mitochondrial electron transport chain involved in oxidative metabolism [22, 30]. Spatial learning has been shown to modulate mitochondrial enzymatic activity and previous work demonstrated that PBM can differentially influence brain metabolic networks depending on their functional engagement, potentially promoting more efficient energy utilization during cognitive processing [43].

Interaction of near-infrared light with CCO enhances electron transport chain activity [5, 41], supporting ATP production and energy-dependent processes such as activity-related gene transcription. In young adult animals, 40 Hz PBM elicit a localized reduction in CCO activity in the prefrontal cortex, the site of stimulation without secondary activation of deeper structures. This finding is consistent with prior evidence indicating that learning-related optimization of neural circuits can be accompanied by reduced sustained metabolic demand [25], reflecting more efficient task-engaged network dynamics rather than diminished neural activation [44]. Importantly, while quantitative CCO histochemistry provides a valuable index of region-specific oxidative metabolic engagement within intact neural circuits, it does not capture all aspects of mitochondrial function, nor transient metabolic fluctuations associated with moment-to-moment neural activity [45, 46].

Metabolic demand during learning is dynamic and evolves across acquisition stages [47]. Early learning phases are typically associated with higher energetic costs due to inefficient network recruitment, whereas later stages rely on more streamlined processing that allows equivalent or improved behavioral performance with lower sustained oxidative demand [46]. In this context, reductions in CCO activity can be interpreted as reflecting learning-related decreases in long-term metabolic load, as previously reported [48], rather than reduced functional engagement.

Interestingly, the relationship between metabolic readouts and behavioral outcomes differed across age groups. In young adults, reduced CCO activity following 40 Hz PBM may reflect increased metabolic efficiency associated with more synchronized and less energetically demanding network activity. In contrast, the absence of detectable CCO modulation in aged animals likely reflects age-related constraints in mitochondrial flexibility or baseline metabolic state, suggesting that PBM-induced cognitive benefits in aging may rely on mechanisms partially independent of measurable changes in oxidative metabolism. Consistent with this view, preclinical studies indicate that PBM can modulate mitochondrial function in an age-dependent manner with chronic stimulation differentially affecting CCO activity and supporting ATP production in aged brains [49–52].

Taken together, these findings suggest that PBM-induced changes in CCO activity reflect adaptive modulation of brain metabolic dynamics under task-engaged and learning- dependent conditions rather than a direct or exclusive marker of enhanced metabolic efficiency [53, 54]. Future studies combining in vivo metabolic mapping with direct assessments of mitochondrial respiration and ex vivo bioenergetic assays will be essential to further delineate the mechanisms underlying PBM-induced functional improvements across the lifespan.

Given that c-Fos activity reflects cellular activation following stimuli application [22], we used the expression of this proto-oncogene to differentiate the effects between PBM stimulation and learning, focusing solely on the impact on various brain regions involved in the previously demonstrated enhanced learning processes such as the PFC and the different layers of the hippocampus. While c-Fos expression serves as a marker for neuronal activation, it cannot differentiate between cell populations or capture specific oscillatory dynamics like gamma-band synchronization [55]. Consequently, it reflects broad activity modulation rather than providing direct evidence of frequency-locked network synchronization [55]. The observed increase in c-Fos in prefrontal CG and IL regions following 40 Hz stimulation, alongside a decrease in hippocampal CA3 under 5 Hz PBM, likely reflects light-induced activation of MAPK/ERK pathways, Ca^2^⁺-dependent transcriptional regulation, and retrograde mitochondrial signaling, in line with photon scattering gradients that distribute energy unevenly across brain regions [22]. Notably, frequency modulation exerted differential effects in the young rats, with 40 Hz producing the strongest enhancement of c-Fos expression at the site of stimulation. Another notable finding is the widespread activation observed across the brain regions studied. The cooperation between the hippocampus and other regions supporting memory processes is well established, with coordinated activity reported during episodic encoding [56–58]. The episodic memory network includes the cingulate cortex [59] and medial PFC [58], highlighting that they belong to an established network. Notably, when transcranial light stimulation targets regions within such intrinsic networks, PBM may enhance activity across the entire network [60]. These results underscore the potential of PBM to modulate neural activity not only locally but also within interconnected circuits critical for memory processes.

PBM appears to exert therapeutic effects without adverse outcomes [36], reducing pro-inflammatory mediators under pathological conditions [61]. In the present study, both 5 Hz and 40 Hz PBM decreased pro-inflammatory markers (IL-1β, IL-6, TNF-α) in the PFC, the site of stimulation. Interestingly, 40 Hz stimulation induced a modest increase in IL-1β, IL-6 and p38 MAPK in the hippocampus, while TNF-α levels remained unchanged.

Importantly, increases in selected cytokines do not necessarily indicate pathological neuroinflammation. Transient and moderate elevations of IL-1β and IL-6 are increasingly recognized as physiological modulators of synaptic plasticity and memory-related processes. PBM is known to induce controlled shifts in cellular redox state and mitochondrial ROS production within physiological ranges [62, 63], activating redox-sensitive transcription factors such as NF-κB [64, 65] and downstream MAPK pathways. These cascades regulate genes involved in cell survival, metabolic adaptation and synaptic remodeling [66] rather than inflammatory damage.

The parallel modulation of IL-6 and p38 MAPK suggests coordinated activation of adaptive stress-response signaling. Such activation may reflect a redox-hormetic mechanism, in which mild oxidative signaling promotes cellular resilience and plasticity. The absence of TNF-α upregulation and the concurrent downregulation of pro-apoptotic markers (p53, BAX) further argue against a maladaptive inflammatory state.

Region-specific cytokine shifts between prefrontal cortex and hippocampus have been reported across diverse learning [67, 68], metabolic [69–71] and aging models [72, 73], supporting the concept of spatially specialized neuroimmune regulation. In this context, the hippocampal cytokine increase following 40 Hz PBM could be understood as a transient, region-specific adaptive signaling response associated with plasticity and network remodeling rather than chronic neuroinflammatory activation. This interpretation is further supported by the concomitant improvement in cognitive performance.

We also observed reductions in pERK and the pERK/ERK ratio across hippocampal subregions at both frequencies. Although PBM can stimulate CCO to increase ATP production and activate the Raf/MEK/ERK pathway [74, 75] our findings suggest a localized activation at the stimulation site with broader inhibitory effects on ERK signaling distally, potentially via homeostatic or neuromodulatory feedback mechanisms.

Anti-inflammatory responses were not uniformly upregulated, as IL-10 decreased under 40 Hz in young animals, while remaining unchanged in aged cohorts animals. IL-10 is critical for dampening pro-inflammatory signaling [76], and prior studies report variable PBM effects depending on context [77]. Importantly, the reduction in IL-10 was not accompanied by an increase in TNF-α expression in the hippocampus, arguing against the establishment of a classical pro-inflammatory state, which typically involves coordinated induction of pro-inflammatory cytokines such as TNF-α and IL-1β [78]. In the absence of overt neuroinflammation or neurodegeneration, the decrease in IL-10 may instead reflect a shift in homeostatic immune tone rather than activation of a pathological inflammatory cascade. Similar context-dependent PBM effects on cytokine modulation have been previously reported [79, 80]. These findings may reflect the absence of neurodegeneration in our models or differences in PBM exposure duration between young and aged cohorts.

Importantly, the observed frequency-dependent modulation of cytokines may be mechanistically linked to mitochondrial and electrophysiological cascades. PBM enhances electron transport chain activity via cytochrome c oxidase, increasing ATP and ROS production, which can activate transcription factors such as NF-κB and p38 MAPK, thereby regulating cytokine expression without inducing pathological inflammation [62]. Furthermore, 40 Hz PBM coincides with gamma oscillations known to synchronize prefrontal–hippocampal networks, influencing microglial activity and supporting homeostatic or adaptive immune signaling [81, 82]. This coordinated neuronal-glial signaling may underline the regional specificity of IL-10 and NF-κB changes, suggesting that PBM frequency can differentially engage neural circuits and microglia to promote plasticity and neuroprotection rather than classical pro-inflammatory responses [83].

Notably, acute 5 Hz PBM increased NF-κB expression in adult animals, a frequency-specific effect not previously reported. Although NF-κB is frequently associated with inflammatory cascades [84, 85], context-dependent activation can promote tissue repair, angiogenesis, and cell survival [86]. By analogy to peripheral tissues, PBM-induced NF-κB in the brain may support neurorepair, neurogenesis, and synaptogenesis, consistent with the observed upregulation of GFAP and synaptic markers. The observed increases in GFAP expression with both 5 Hz and 40 Hz frequencies indicate the PBM potential to enhance synaptic homeostasis and provide neuroprotection. Specifically, in the hippocampus our results showed that higher frequencies may provide enhanced support for synaptic remodeling during memory and learning processes. These results suggest that specific PBM frequencies can differentially engage NF-κB to facilitate adaptive neural responses.

To determine whether different frequency patterns of PBM could produce changes in neurons or glial cells, we performed immunoblots to quantify their expression levels. We also probed the immunoblots for pre-synaptic marker (Synapsin-I) and post-synaptic marker (PSD-95) as general markers for synaptogenesis. PBM induces frequency-dependent changes in synaptic proteins, with 40 Hz producing greater increases in Synapsin-I and PSD-95 expression in the place of application, such as the PFC, while both 5 Hz and 40 Hz enhance synaptic protein levels in the hippocampus suggesting the capacity of both PBM frequencies to propagate its action across widespread neural circuits.

Finally, cell death pathways also exhibit proinflammatory characteristics, which may contribute to the aging process [87]. We tested our hypothesis that PBM can improve mitochondrial apoptosis by modulating the p53/BCL-2/BAX interaction. Our findings indicated that 40 Hz frequency PBM has indeed the potential to modulate -apoptosis-related signaling. BCL-2 family proteins and the tumor suppressor protein p53 are key regulators of apoptosis signaling pathways [88, 89]. p53 modulates the activity of the BCL-2 family through both transcription-dependent and transcription-independent mechanisms [90, 91]. The mitochondrial apoptosis pathway, also known as the intrinsic apoptosis pathway, is tightly regulated by the pro-apoptotic and anti-apoptotic members of the BCL-2 family through intricate protein‒protein interaction networks [92]. The direct interactions between p53 and the anti-apoptotic proteins BCL-2 and BCL-xL play critical roles in the p53-mediated regulation of mitochondrial apoptosis [90, 93, 94]. Both p53 and the pro-apoptotic protein BAX were downregulated in PFC, a region not directly irradiated. This aligns with structural models of the p53 DNA-binding domain complexed with BCL-2 [95] and prior evidence of PBM-mediated suppression of mitochondrial-dependent neuronal apoptosis [96]. By reducing p53 expression, PBM may limit competition with BAX for BCL-2 binding, shifting the balance toward anti-apoptotic signaling and contributing to broader neuroprotection.

In the aging cohort, PBM was applied exclusively at 40 Hz, a frequency selected based on the behavioral and neurobiological effects observed in adult animals, as well as its established relevance to gamma-band cognitive processes [81, 97]. While this design allowed us to test the efficacy of the most promising stimulation pattern in aging, the absence of a 5 Hz condition in aged animals limits the ability to directly assess frequency specificity in later life. Accordingly, conclusions regarding frequency-dependent effects across the lifespan should be interpreted with caution and are restricted to demonstrating the efficacy of 40 Hz PBM in aged animals.

Collectively, these findings highlight frequency and region-specific effects of PBM on neural activation, inflammatory signaling, and apoptosis, providing mechanistic insight into its capacity to enhance cognition and promote neuroprotection in both young and aged brains. These findings also showed that PBM treatment at 40 Hz not only supports the consolidation and retrieval of spatial memory, which are typically impaired in aging but also enhances adaptive cognitive processes, allowing older subjects to update learned information more efficiently.

## Limitations

Several limitations of this study should be considered. First, while our findings highlight the potential relevance of 40 Hz PBM for age-related cognitive decline, several translational constraints exist. Human cranial anatomy limits light penetration, with only ~ 1–2% of NIR light (660–940 nm) reaching cortical tissue, in contrast to much higher transmittance in rodents (21–54%, depending on cranial thickness) [98, 99]. Additional interspecies differences in cortical thickness, network organization, and the lack of long-term human data on repeated PBM sessions warrant caution when extrapolating these results beyond the preclinical context.

Second, although group sizes were consistent with prior behavioral PBM studies [43, 100], larger cohorts may be necessary to detect subtle molecular effects and refine effect size estimates. The absence of a 5 Hz comparison group in aged animals also precludes definitive conclusions on frequency-specific effects across the lifespan.

Finally, the study was conducted exclusively in male rats. Given known sex-dependent differences in aging trajectories, neuroinflammatory responses, and neuromodulatory sensitivity, the exclusion of females limits generalizability and underscores the need for future studies incorporating both sexes to more fully define PBM’s therapeutic potential in aging.

## Data Availability

The data that support the findings of this study are available from the corresponding author upon reasonable request.
